# Characterization of the first highly predatory *Bdellovibrio bacteriovorus* from Iran and its potential lytic activity against principal pathogenic *Enterobacteriaceae*

**DOI:** 10.22038/ijbms.2020.43159.10146

**Published:** 2020-10

**Authors:** Salman Odooli, Rasoul Roghanian, Giti Emtiazi, Milad Mohkam, Younes Ghasemi

**Affiliations:** 1 Department of Cell and Molecular Biology and Microbiology, Faculty of Biological Sciences and Technology, University of Isfahan, Isfahan, Iran; 2 Pharmaceutical Sciences Research Center, Shiraz University of Medical Sciences, Shiraz, Iran; 3 Department of Pharmaceutical Biotechnology, School of Pharmacy, Shiraz University of Medical Sciences, Shiraz, Iran

**Keywords:** 16S rRNA Analysis, Bacteriolytic activity, Bdellovibrio, Enterobacteriaceae, Iran, Isolation, Predation, Transmission electron- microscopy

## Abstract

**Objective(s):**

*Bdellovibrio*-and-like organisms (BALOs) are predatory prokaryotes that attack and kill other Gram-negative bacteria for growth and reproduction. This study describes the isolation, identification, biological properties, and bacteriolytic activity of the first *Bdellovibrio* *bacteriovorus* with a broad prey range from Iran.

**Materials and Methods:**

One BALO strain with high predatory potency was isolated from the rhizosphere soil using Enteropathogenic *Escherichia coli* as prey. It was identified and designated as *Bdellovibrio bacteriovorus* strain SOIR-1 through plaque assays, transmission electron microscopy (TEM), *Bdellovibrio*-specific PCRs, and 16S rRNA gene sequence analysis. Biological characterization and analysis of bacteriolytic activity were also performed.

**Results:**

TEM and *Bdellovibrio*-specific PCRs confirmed that the strain SOIR-1 belongs to the genus *Bdellovibrio*. Analysis of the 16S rRNA gene sequence revealed its close phylogenetic relationship with strains of *Bdellovibrio bacteriovorus*. The strain SOIR-1 grew within the temperature range of 25–37 ^°^C and the pH range of 6.0–8.0, with the optimal predatory activity at 30 ^°^C and pH 7.4. It had the highest and lowest bacteriolytic activity toward *Shigella dysenteriae* and *Pseudomonas aeruginosa* with a killing rate of 89.66% and 74.83%, respectively.

**Conclusion:**

Considering the hypothesis of bdellovibrios heterogeneity, identification of new isolates contributes to a deeper understanding of their diversity, their ecological roles, and their promising potential as living antibiotics or biocontrol agents. Bdellovibrios with broad bacteriolytic nature has not previously been reported in sufficient detail from Iran. The results of this study showed the great potential of native *B. bacteriovorus* strain SOIR-1 in the control and treatment of diseases caused by pathogenic *Enterobacteriaceae*.

## Introduction

There is an increasing concern about the emergence of drug resistance within bacterial communities following the extensive use and abuse of antimicrobial agents. Distribution of genetic determinants associated with antibiotic resistance among bacterial communities has led to the development of multidrug-resistant (MDR) pathogenic bacteria, the inefficiency of existing antimicrobial agents, and the gradual disarmament of humans against bacterial infections (1-3). Another challenge is bacterial biofilms that are up to 1000 times more resistant to antimicrobial agents than their planktonic counterparts, even without genetic markers required for antimicrobial resistance (4-6). Predatory prokaryotes have recently been suggested as one of the alternative therapeutic strategies (7-9). They are smaller than their prey and exist as a living organism compared with other predatory microorganisms (protists and bacteriophages). Predatory bacteria acquire their required biosynthetic materials and energy from other live bacterial cells as prey (8, 10, 11).

The predatory mechanisms of predatory bacteria are divided into four main categories based on the nature of the interactions between the predator and prey. *Myxococcus* and *Lysobacter *employ the wolf-pack or group predation approach in which numerous predator cells produce and secrete a variety of hydrolytic enzymes for degrading the encircled bacterial prey cell, without any physical attachment to the prey cells. In the epibiotic predation scenario, *Vampirococcus* attaches to the outer surface of the *Chromatium* prey cell and begins to degrade and assimilate host molecules through specialized structures between the predator and its prey. *Daptobacter* uses the endobiotic predation mechanism in which the predator cell enters into the prey cytoplasm, degrades and consumes the prey components, and then lyses the prey cell. Almost all *Bdellovibrio-*and-like organisms (BALOs) predate through the periplasmic invasion approach in which the predator cell exclusively invades and enters the periplasmic space of Gram-negative prey bacterium, where it grows at the expense of the prey macromolecules. It seems that only wild-type BALOs are obligate predators. Other predatory bacteria can grow heterotrophically and multiply in the absence of prey cells (8, 11).

BALOs taxonomically belong to the class *δ-proteobacteria* and order* Bdellovibrionales*, and are grouped into three families; *Bdellovibrionaceae* (terrestrial bdellovibrios), *Bacteriovoraceae* (marine halophilic *Bacteriovorax*), and *Peridibacteraceae* (12, 13). BALOs are ubiquitous in natural ecosystems and have been isolated from biotic and abiotic niches (10, 12, 14-16). *Bdellovibrio bacteriovorus*, the most well-known BALOs, was accidentally discovered by Stolp and Petzold in 1962 during the isolation of soil bacteriophages for biocontrol of phytopathogenic* Pseudomonas syringae* pv. *phaseolicola*. Bdellovibrios are small (about 0.2-0.5 μm wide and 0.5-2.5 μm long) vibrio-shaped Gram-negative cells, with a single sheathed polar waveform flagellum (9, 10, 17, 18). The genome and proteome composition of *B. bacteriovorus *were determined and analyzed by Rendulic *et al*. and Pan *et al*. (19, 20). It’s not strange for an obligate predator like* B. bacteriovorus *to have too many potential genes encoding molecular arsenals and hydrolytic enzymes. Although the mechanism of action of these enzymes is not yet fully known, their study helps to understand the predation mechanism. Some of these enzymes can also be used as the foundation for the development of future therapeutic agents (11, 17).

**Figure 1:**
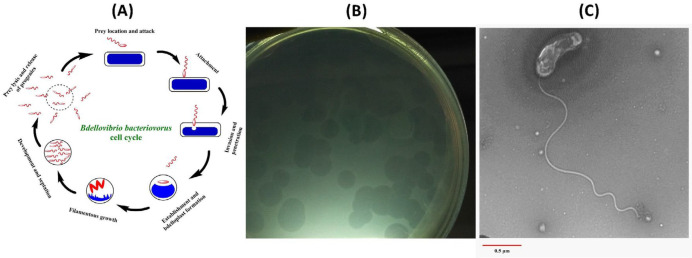
(A) The predatory life cycle of *Bdellovibrio bacteriovorus*. (B) Typical lytic plaques developed by *B. bacteriovorus* strain SOIR-1 on the lawn of *Escherichia coli* prey cells. (C) Microscopic identification of isolated *B. bacteriovorus* strain SOIR-1 in the attack phase using a transmission electron microscope (TEM). The approximate body size is 0.8 μm in length, 0.25 μm in width, and the flagellum length is 2.1 μm. The scale bar represents 0.5 μm

The life cycle of *B. bacteriovorus* is biphasic (Figure 1A). *Bdellovibrio* searches for prey in the free-swimming attack phase, attaches to the outer membrane of the prey cell, detaches its flagellum, and penetrates the prey’s periplasmic space using a set of hydrolytic enzymes. The prey cell metabolism is deactivated in the early intraperiplasmic growth phase. The infected prey cell is transformed into a globular structure called “bdelloplast”, where the invading *Bdellovibrio* inserts hydrolytic enzymes into the prey’s cytoplasm and utilizes prey macromolecules as a source of nutrients and energy for reproduction. The progenies of *Bdellovibrio* are synthesized in the form of a filamentous and nonseptated structure, which eventually divides into individual flagellated motile progeny cells upon depletion of prey’s cytoplasm. Finally, the bdelloplast is lysed, and mature attack phase *Bdellovibrio* cells are released to resume new life cycles (13, 17, 20-22). 

Bdellovibrios possess a unique genetic locus called *hit *(host interaction) that encodes proteins necessary for attachment and invasion of the prey cell. Some *Bdellovibrio *derivatives have been developed through very-low-frequency mutations in the *hit *locus. These prey (host)-independent derivatives can grow on the nutrient-rich media in the absence of live prey cells. They also have different morphological and physiological properties (especially predatory potency) compared with wild-type prey (host)-dependent bdellovibrios (13, 22, 23).

The interaction between bdellovibrios and their prey cells can be considered a parasite-host relationship, i.e., invading a substrate cell for reproduction. However, this interaction can be a predator-prey relationship because bdellovibrios do not use the prey’s metabolic machinery but directly digest the prey cell as a substrate to provide their required monomers (10). Bdellovibrios are also capable of penetrating and predating inside biofilms. The bacteriolytic nature of bdellovibrios against the human, animal, plant, and aquatic Gram-negative pathogens regardless of their metabolic and antimicrobial resistance status, make them attractive candidates as potential living antibiotic and biocontrol agents (7, 9, 16, 17, 24-29).

In this study, we presented the first complete documentation on the isolation, molecular identification, and biological characterization of a highly bacteriolytic *Bdellovibrio* strain from Shiraz city in Southwestern Iran, which has not been fully reported in sufficient detail in this country before. This study will potentially add to the existing database on bdellovibrios and will help to bridge the gap between the research findings on BALOs in other countries. The presented data could also confirm the possible potential of the isolated *Bdellovibrio *strain as a living antibiotic against principal pathogenic *Enterobacteriaceae*.

## Materials and Methods

***Prey preparation***

The bacterial strains used in this study are listed in Table 1. Enteropathogenic *Escherichia coli* (PTCC 1270) was used as prey for preliminary isolation of BALOs. All bacterial strains were cultured overnight in the Tryptic soy broth (Merck, Darmstadt, Germany) supplemented by 0.2% yeast extract (HiMedia, Mumbai, India) at 37 ^°^C with shaking at 160 rpm until the end of exponential to early stationary phase. The prey cells were then harvested by centrifugation (6500 ×g for 15 min at 4 ^°^C), and the resultant pellets were washed twice and re-suspended in the sterile HM buffer containing 25 mM HEPES (4-[2-hydroxyethyl]-1-piperazineethanesulfonic acid) (Fisher Scientific, Fair Lawn, New Jersey, USA) supplemented by 0.22-μm pore size filter-sterilized (JET-BIOFIL, Guangzhou, China) 3 mM CaCl_2_,2H_2_O and 2 mM MgCl_2_,6H_2_O, final pH 7.4. The prey cell suspensions were then adjusted to an optical density of 2.0 at 600 nm (OD_600_) [~10^9^ colony forming units per ml (CFU/ml)] using a spectrophotometer apparatus (Eppendorf, Hamburg, Germany).

***Sample collection, processing, and enrichment for BALOs***

The rhizosphere soil sample was collected from agricultural land (located in the School of Pharmacy, Shiraz, Iran; longitude 52°33’ E, latitude 29°41’ N) using a clean hand shovel and after removing about 10 cm of top soil. The temperature of rhizosphere soil was determined *in situ* using a portable digital thermometer. The sample was poured into a sterile polythene bag and transferred immediately to the laboratory for subsequent analysis. Five scoops of sterile distilled water were added to the five scoops of soft soil, and the mixture was stirred for 20 sec. The soil pH was measured after 15 min using a digital pH meter. A slurry mixture was prepared in a sterile 1000 ml Erlenmeyer flask by suspending 35 g rhizosphere soil sample in 350 ml sterile HM buffer. The mixture was agitated on an orbital shaker (200 rpm for 1 hr at room temperature) to ensure the loosening of soil clumps and the uniform distribution of organisms. Afterward, the slurry mixture was enriched by 30 ml of washed* E. coli *prey cell suspension (prepared as described above) and incubated for 72 hr at 30 ^°^C on an orbital shaker at 200 rpm.

***BALO isolation***

The slurry mixture was subjected to the sequential centrifugation at 800 ×g, 1800 ×g, and 4500 ×g for 15 min at 4 ^°^C to remove large particles and most microorganisms. The resultant supernatant was filtered three times through a 0.45-μm pore size syringe filter (Orange scientific, Braine-l’Alleud, Belgium) for trapping the remaining bacteria and debris. The filtrate was centrifuged at 27000 ×g for 20 min at 4 ^°^C, which efficiently concentrates the possible BALOs cells in the pellet and eliminates potential bacteriophages in the supernatant. The resultant pellet was washed and re-suspended in 2 ml HM buffer and then serially diluted 10-fold up to 10^−10^ with HM buffer. These dilutions were checked for viable BALOs using the double-layer agar plating technique as follows: 300 μl of each dilution was mixed with 900 μl of washed prey cell suspension (*E. coli*, ~10^9^ CFU/ml) in 6 ml of molten HM top agar (25 mM HM buffer amended with 3 mM CaCl_2_, 2 mM MgCl_2_, 0.7% agar, final pH 7.4), and the mixture was then spread over the HM bottom agar plates (25 mM HM buffer amended with 3 mM CaCl_2_, 2 mM MgCl_2_, 1.5% agar, final pH 7.4). The plates were incubated at 30 ^°^C, and plaque formation was monitored for 10 days. Small and clear lytic regions that developed within 24 hr but did not expand further were considered as probable bacteriophages. The potential BALOs plaques were screened through the following features: round and regular with sharp boundaries, no colonies at their centers, development after 3-5 days, and progressive increase in size. Such plaques were lifted from the plate, re-suspended in 4 ml HM buffer, and checked for small comma-shaped cells with high motility using phase-contrast microscopy. One of the best plaque suspensions was selected, passed three times through a 0.45-μm filter, and purified using three successive double-layer agar plating procedures, as described above.

***Preparation and storage of attack-phase BALO***

A plug of fresh BALO plaque and the surrounding prey was lifted from the double-layer agar plate and inoculated into 25 ml of washed* E. coli* prey cell suspension (~10^9^ CFU/ml). The co-culture was incubated at 30 ^°^C with shaking at 200 rpm and monitored for lysis of prey cells (clearing the prey suspension and reduction of initial OD_600_) within 72 hr (stock lysate). The remaining prey cells were eliminated by centrifugation (5000 ×g for 20 min at 4 ^°^C) and filtration of resultant supernatant (0.45-μm) (filtrate lysate). Filtrate lysate containing attack-phase BALO was concentrated by centrifugation at 27000 ×g for 20 min at 4 ^°^C. The resultant pellet was then re-suspended in HM buffer and adjusted to ~10^9^ plaque-forming units per ml (PFU/ml) through optical density measurement (10^9^ PFU/ml gave OD_600_ nm ca. 0.15). The isolated BALO was stored as pure plaques or stock lysates for up to 1 month at 4 ^°^C. For long-term storage, aliquots of fresh stock lysate were mixed with 80% glycerol to a final concentration of 20% (v/v). The mixtures were quickly frozen in liquid nitrogen and stored up to 4 years at -80 ^°^C.

***BALO identification***

*Transmission electron microscopy (TEM)*

One drop of freshly prepared attack-phase BALO suspension was placed on a Formvar carbon-coated 300-mesh copper microscope grid for 5 min at room temperature. The grid was then lifted gently with a pair of forceps, and the excess liquid was removed. The sample was then counterstained with 1% (w/v) solution of uranyl acetate (pH 4.0) for 10 min and examined with a Philips CM10 transmission electron microscope at an accelerating voltage of 100 kV (Laboratory of transmission electron microscopy, School of Veterinary Medicine, Shiraz University, Shiraz, Iran). Small and vibrioid-shaped cells (0.25 to 0.40 μm in width, 1 to 2 μm in length) with one long sheathed polar flagellum are considered as potential attack-phase BALOs.

*Analysis of partial 16S rRNA gene*

Genomic DNA of attack-phase BALO (filtrate lysate, ~10^9^ PFU/ml) was extracted using the CinnaPure-DNA Kit for Gram-negative bacteria (SinaClon, Tehran, Iran), and its concentration was adjusted to 20 ng/µl using a Pico200 picodrop spectrophotometer (Picodrop Ltd, Hinxton, United Kingdom). For phylogenetic analysis, the 16S rRNA gene was PCR amplified using the universal primers (Table 2) (30) in a total reaction volume of 40 μl containing: 20 µl Taq DNA polymerase master mix red 2X (Ampliqon, Odense, Denmark), 1 µl of each primer (20 µM), 3 µl template DNA, and 15 µl sterile deionized distilled water. Amplifications were performed using a DNA thermo-cycler (BIO-RAD, Hercules, CA, USA) with the following PCR conditions: Initial template denaturation step at 94 ^°^C for 4 min, followed by 35 cycles of denaturation at 94 ^°^C for 1 min, annealing at 50 ^°^C for 1 min, elongation at 72 ^°^C for 1 min, and the final extension step at 72 ^°^C for 5 min. Successful amplifications were confirmed through electrophoresis of PCR products on 1.8% Tris-Borate-EDTA (TBE) agarose gel and visualized using a U:Genius3 GelDoc system (SYNGENE, Cambridge, United Kingdom). The desired amplicon (∼1300 bp) was extracted and purified using a Gel Purification Kit (Bioneer, Daejeon, South Korea) and sent for bidirectional sequencing (Macrogen, Seoul, South Korea). The obtained sequences were modified using BioEdit (version 7.1.9) and Chromas Pro (version 2.1.3) programs. The final 16S rRNA gene sequence was then searched for similarity in the NCBI databases using the BLAST-N search tool. The 16S rRNA gene sequences of the representative strains (Supplementary Table 1) were retrieved from the GenBank and then aligned using the MUSCLE program, which is implemented in the MEGA software package. A phylogenetic tree was constructed through the Neighbour-Joining algorithm (p-distance model) using MEGA software version 7.0.26 with a bootstrap analysis of 1000 replicates (31).

***Bdellovibrio-specific PCR reactions***

Specific 16S rRNA gene amplification was performed using the *Bdellovibrio*-specific 16S rRNA primers (14) (Table 2) under the following PCR conditions: initial template denaturation step at 95 ^°^C for 5 min, followed by 25 cycles of denaturation at 95 ^°^C for 1 min, annealing at 53 ^°^C for 1 min, and elongation at 72 ^°^C for 1 min. The final extension step was 10 min at 72 ^°^C. The *hit* locus was also amplified using the specific primer pairs (32) (Table 2) under the following PCR conditions: initial template denaturation step at 95 ^°^C for 5 min, followed by 20 cycles of denaturation at 95 ^°^C for 1 min, annealing at 60 ^°^C for 1 min, and elongation at 72 ^°^C for 1 min. The final extension step was 10 min at 72 ^°^C. The* E. coli* genomic DNA served as the negative control in the specific PCR reactions, and the successful amplifications were confirmed by agarose gel electrophoresis (1.8%) and visualized using the GelDoc apparatus.

***Characterization of Bdellovibrio bacteriovorus strain SOIR-1 bacteriolytic activity***

Two separate methods evaluated the lytic activity of strain SOIR-1 against several prey bacteria (Table 1). First, the formation of clear lytic plaques on the lawn of prey cells using the double-layer agar plating technique. Second, lysis analysis in the broth co-cultures followed by the reduction of OD_600_ and CFU/ml of prey cell suspensions and mutual increase in the cell density (PFU/ml) of strain SOIR-1. In the plaque formation assay, the fresh filtrate lysate of attack-phase strain SOIR-1 (~10^9^ PFU/ml) was serially diluted 10-fold, and 300 µl of each dilution was mixed with 900 µl of each washed prey cell suspension (~10^9^ CFU/ml) in 6 ml of molten HM top agar. The mixture was immediately spread over the surface of HM bottom agar, and the plates were incubated for 10 days at 30 ^°^C. For lysis analysis in the broth co-culture, 25 ml of each washed prey cell suspension (~10^9^ CFU/ml) was inoculated with 400 µl of pure strain SOIR-1 in the attack-phase (final cell density of ~10^4^ PFU/ml). The co-cultures were incubated for 7 days at 30 ^°^C with shaking at 200 rpm. The CFU/ml (using standard spread-plate count), PFU/ml (through described double-layer agar plating method), and OD_600_ parameters were monitored at 24 hr intervals. The non-inoculated prey cell suspensions, heat-killed (95 ^°^C, 15 min) prey cell suspensions inoculated with strain SOIR-1, prey cell suspensions inoculated with filtered strain SOIR-1 (0.22-μm pore size), and strain SOIR-1 cell suspension without any prey served as negative controls. Killing rate (%) and ΔOD_600_ were calculated for each prey through the following formulas:

$∆\mathrm{OD}={\mathrm{OD}}_{600}\left(s\right)-{\mathrm{OD}}_{600}\left(\mathrm{Se}\right)$

$\mathrm{Killing rate}=[\frac{N_{0}-N_{i}}{N_{0}}]\times 100$

where OD_600_ (s) and OD_600_ (e) are the OD_600_ at the starting day and day e (evaluation day), respectively. Similarly, N_0_ and N_i_ are the numbers of bacterial prey cells at day 0 (starting day) and day i (evaluation day), respectively. 

***Effects of temperature and pH on the predation by Bdellovibrio bacteriovorus strain SOIR-1***

The evaluation of temperature effect was achieved by inoculating 25 ml of washed *E. coli* prey cell suspensions (~10^9^ CFU/ml, pH 7.4) with 400 µl of strain SOIR-1 in the attack-phase (final cell density of ~10^4^ PFU/ml). The co-cultures were incubated for 7 days at 200 rpm, with temperatures set at 25, 30, 35, 37, and 42 ^°^C. The changes in the cell number of strain SOIR-1 (PFU/ml) and *E. coli* prey (OD_600_ and CFU/ml) in the co-cultures were monitored at 24 hr intervals. The non-inoculated prey cell suspensions served as negative controls. The pH effect was examined at 30 ^°^C using HM buffers with different pH (5.5, 6.0, 6.5, 7.0, 7.4, 8.0, and 8.5) in the same way as mentioned for temperature effect.

***Statistical analysis***

All experiments were carried out in triplicate, and each experiment was repeated three times. The results were presented as the mean±standard deviation (SD) error. The analysis of variance (ANOVA) and *Post hoc* Tukey’s test were used for multiple comparisons between groups (GraphPad Prism v 6.07 for Windows, GraphPad Software, La Jolla, California, USA). Differences were considered significant at *P*-value≤0.05 level. The drawing of graphs and diagrams was done by the GraphPad Prism v 6.07 software and Microsoft Office for Windows^©^ tools, 2013 (Microsoft, Redmond, Washington, USA). Other mathematical and statistical analyses were performed using Microsoft Excel^©^ for Windows^©^.

## Results

***BALO isolation***

The rhizosphere soil sample had a moist dark-brown loamy texture with a temperature of 28 ^°^C and pH 7.1. It was initially enriched with *E. coli* to saturate the potential BALOs for easier observation by subsequent plaque-based assays. Several lytic regions were developed on the lawn of *E. coli* prey cells in double-layer agar plates after 5 days of incubation at 30 ^°^C. One of the largest plaques was selected for subsequent analysis and considered as a potential BALO through plaque properties and phase-contrast microscopic verifications. Following three purification steps, this plaque became visible after 72 hr of incubation at 30 ^°^C (Figure 1B). The size of plaques depended on the concentration of the BALO inoculated into the bottom agar plates and reached 4.0–9.0 mm after 10 days of incubation at 30 ^°^C (Supplementary Figure 1). These plaques expanded upon more incubation time and eventually covered almost the entire lawn of *E. coli* prey in double-layer agar plates (Supplementary Figure 1). The growth of plaques is due to the movement of the predator within the soft (top) agar. This is a prominent feature of BALOs and distinguishes them from bacteriophages (18, 33). Phase-contrast microscopy revealed that the isolated predators were small comma-shaped cells with high motility. All of these findings confirm that the isolated predator presumably belongs to the BALOs. 

***BALO identification and phylogenetic analysis***

**Figure 2:**
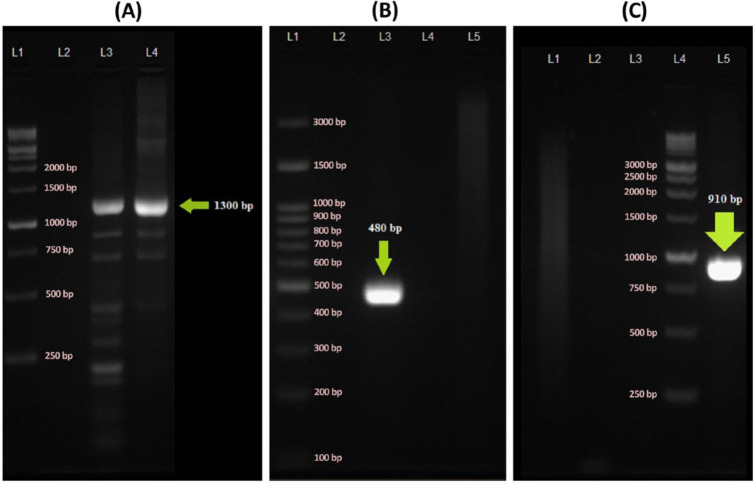
(A) Agarose gel electrophoresis of PCR assay using universal 16S rRNA gene primers (63F-1378R). L1; DNA ladder marker (1k bp), L2; Distilled water negative control, L3; *Escherichia coli* genomic DNA, L4; *Bdellovibrio bacteriovorus* strain SOIR-1 genomic DNA. (B) Agarose gel electrophoresis of PCR assay using *Bdellovibrio*-specific 16S rRNA gene primers (Bd529F-Bd1007R). L1; DNA ladder marker (100 bp), L2; Empty well, L3; *B. bacteriovorus* strain SOIR-1 genomic DNA, L4; Distilled water negative control, L5; *E. coli* genomic DNA. (C) Agarose gel electrophoresis of PCR assay using *Bdellovibrio*-specific *hit locus* primers (Hit FW-Hit RW). L1; *E. coli* genomic DNA, L2; Distilled water negative control, L3; Empty well, L4; DNA ladder marker (1k bp), L5; *B.* *bacteriovorus* strain SOIR-1 genomic DNA

Transmission electron microscopy (TEM) revealed that the isolated BALO was a small vibrioid-shaped cell about 0.8 μm long and 0.25 μm wide, which had a single polar sheathed flagellum of 2.1 µm in length (Figure 1C). These morphological characteristics were striking and distinctive features of bdellovibrios (12, 18). For further identification, the 16S rRNA gene was amplified using PCR with the universal primers (63F-1378R), and the obtained amplicon (~1300 bp, Figure 2A) was sequenced bidirectionally. One contig (1192 bp) was generated through the modification and complementation of the two obtained sequences.

The BLAST-N search indicated that the isolated BALO had 98-99% similarity (Query Covers of 100%) to the strains of *B. bacteriovorus*, including DM7C, HD100, JSF1, SRE7, angelus, and SSB218315 (GenBank accession numbers of KU973530.1, NR_027553.1, EU884925.1, AF263832.1, GQ427200.1, and KT807464.1, respectively). The isolated BALO showed the minimum similarity (92%, Query Covers of 100%) to the FFRS-5 and JSS strains of *Bdellovibrio* *exovorus* (GenBank accession numbers of KP272154.1 and NR_102876.1, respectively) (Supplementary Table 1). Accordingly, the isolated BALO was considered a strain of *B. bacteriovorus* and designated as strain SOIR-1. The 16S rRNA gene sequence obtained in this study (1192 bp) was deposited in the GenBank database under the accession number of MG230309.1 for *B. bacteriovorus *strain SOIR-1.

Two distinct *Bdellovibrio*-specific PCR amplification assays targeting the 16S rRNA gene (Figure 2B) and *hit* locus (Figure 2C) were also performed to strengthen the results. The expected PCR products were generated only when the genomic DNA of strain SOIR-1 served as the template. Successful amplification of* hit* locus further confirms that the isolated strain SOIR-1 belongs to the *B.* *bacteriovorus *since it has been proposed that the *hit *locus is restricted to *B. bacteriovorus *(15).

**Figure 3:**
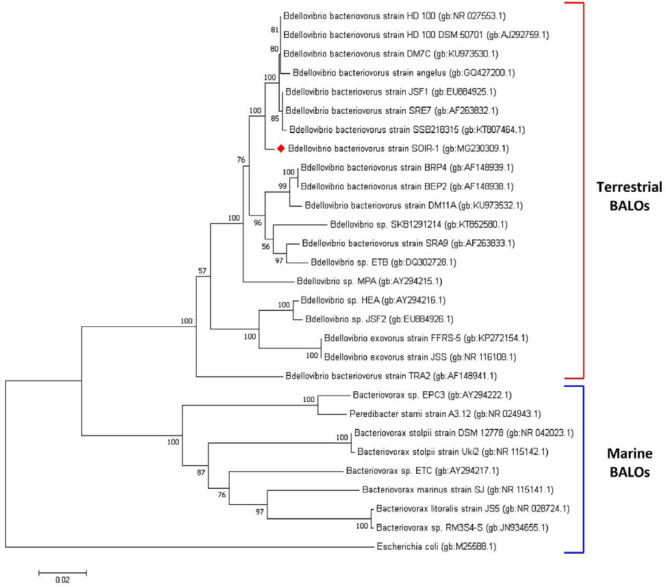
The phylogenetic tree based on the 16S rRNA gene sequences of BALOs. The tree was constructed using the Neighbour-Joining algorithm and the p-distance model. Bootstrap confidence was calculated from 1000 replicates. The *Escherichia coli* 16S rRNA gene sequence was used as an external group

The phylogenetic tree constructed based on the 16S rRNA gene sequences (Figure 3) showed that the strain SOIR-1 could be a new strain since it first formed an independent branch and then clustered with the other group of soil-associated strains of bdellovibrios. The marine and terrestrial BALOs were initially grouped as family* Bdellovibrionaceae*. They were then divided into two distinct families based on the G+C content, prey preference, and response to salinity. There is a separate phylogenic relationship between the marine (family *Bacteriovoraceae*) and terrestrial (family *Bdellovibrionaceae*) BALOs (16, 34). This concept is quite evident in the phylogenetic tree of strain SOIR-1.

***Bacteriolytic potency of Bdellovibrio bacteriovorus strain SOIR-1***

The bacteriolytic ability was investigated through the plaque formation assay (Table 1) and monitoring of lysis parameters (OD_600_, CFU/ml, and PFU/ml) in the broth co-cultures. The strain SOIR-1 was able to lyse all live Gram-negative prey (Supplementary Figure 2). A significant decrease in OD_600_ and CFU/ml occurred in the co-cultures of live Gram-negative preys inoculated with strain SOIR-1 (*P*-value≤0.05). However, OD_600_ and CFU/ml did not change remarkably in the case of non-inoculated preys with strain SOIR-1, preys inoculated with filtered strain SOIR-1 (0.22-µm), and heat-killed preys inoculated with strain SOIR-1 (*P*-value>0.05) (Supplementary Figures 2A-F). The inability of bdellovibrios to pass through the 0.22-µm pore size filter distinguishes them from bacteriophages. Furthermore, there was no live host cell to support the growth of strain SOIR in the treatments containing heat-killed prey. So, the strain SOIR-1 is a prey-dependent (wild-type) *Bdellovibrio.* No plaque was also developed regarding the mentioned negative controls. 

In the co-cultures of live Gram-negative preys, at the same time as the number of prey cells (CFU/ml) decreased significantly (*P*-value≤0.05), the cell density of strain SOIR-1 (PFU/ml) increased significantly (*P*-value≤0.05) (insets in Supplementary Figures 2A-F). The strain SOIR-1 failed to lyse Gram-positive bacteria. There was no considerable change in OD_600_, CFU/ml, and PFU/ml parameters regarding the co-cultures of Gram-positive preys (*P*-value>0.05) (Supplementary Figure 3). All these specifications were matched with the characteristics of bdellovibrios. Furthermore, the prey range analysis reaffirms that the isolated strain SOIR-1 belongs to bdellovibrios since bdellovibrios have a broader prey range than bacteriophages.

**Figure 4:**
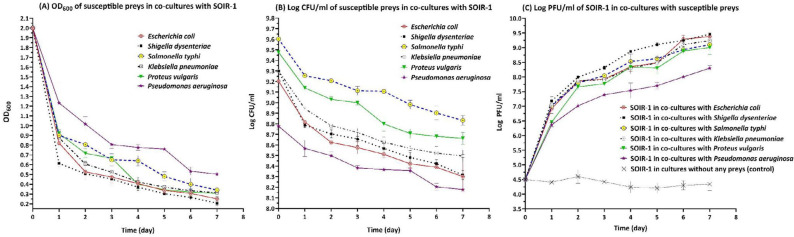
OD_600_ (A), CFU/ml (B), and PFU/ml (C) parameters in the broth co-cultures of susceptible preys with *Bdellovibrio* *bacteriovorus* strain SOIR-1

The sharpest changes in OD_600_, CFU/ml, and PFU/ml occurred within the first 24-48 hr (*P*-value≤0.05), and then the intensity and slope of these changes became milder (*P*-value>0.05) (Figures 4A-C).

**Figure 5:**
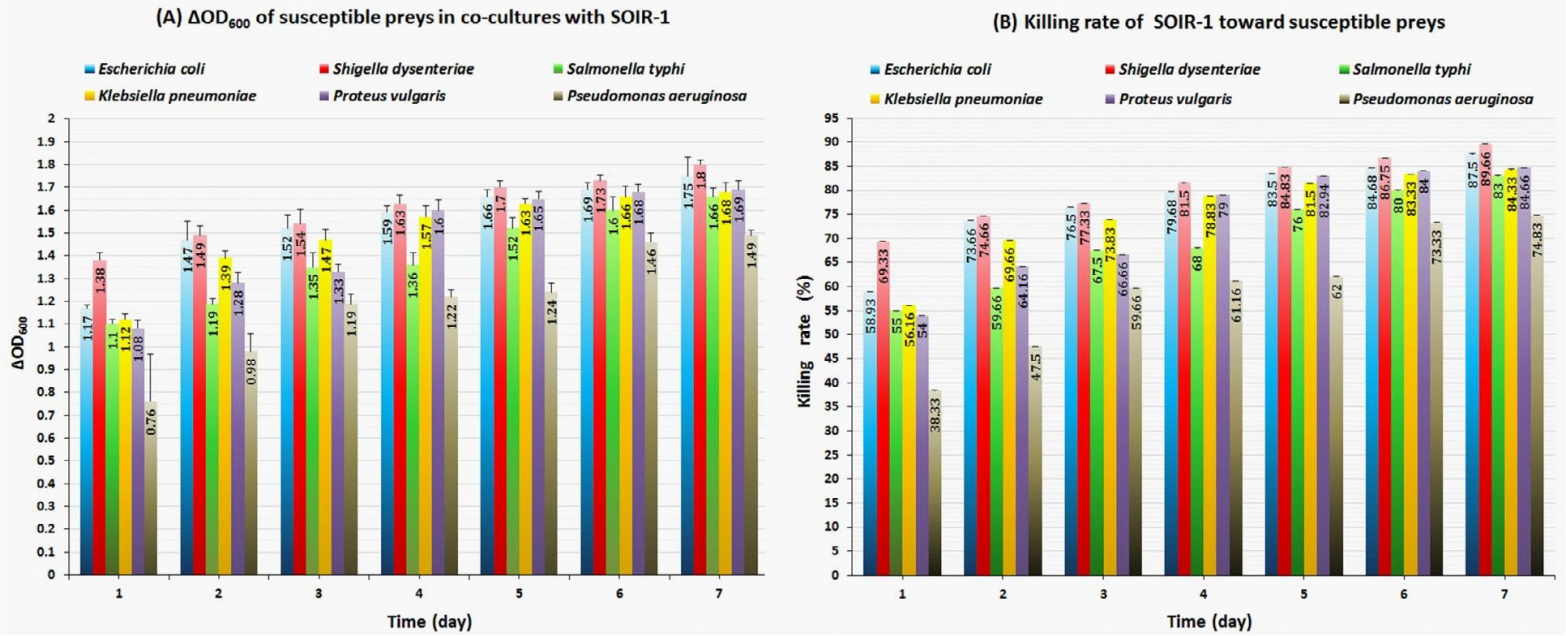
ΔOD_600_ and killing rate parameters in the broth co-cultures of *Bdellovibrio bacteriovorus* strain SOIR-1 with preys

The OD_600 _reduction rate (ΔOD_600_) of *Shigella dysenteriae* was higher than other preys, and *Pseudomonas aeruginosa* had the lowest OD_600_ reduction rate throughout the 7 days of analysis. In the first 24 hr, the reduction rate in OD_600_ of* S. dysenteriae* and *P. aeruginosa* were 1.38±0.03 and 0.76±0.2, respectively. At the end of the evaluation period (day 7), the highest OD_600 _reduction rate was observed for *S. dysenteriae* (1.8±0.02), and *P. aeruginosa *had the lowest one (1.5±0.02) (Figure 5A). Mutually, the highest and lowest reduction in log CFU/ml was observed for *S. dysenteriae* and *P. aeruginosa*, respectively (Supplementary Figure 4A). The highest reproduction rate of strain SOIR-1 (PFU/ml) was associated with the most considerable reduction in the number of prey cells (CFU/ml) (Supplementary Figure 4B). The killing rate (%) of strain SOIR-1 toward susceptible preys at the end of the assessment period (day 7) was as follows (from the highest to lowest):* S. dysenteriae* (89.66%), *E. coli* (87.5%), *Proteus vulgaris* (84.66%), *Klebsiella pneumonia* (84.33%), *Salmonella typhi* (83%), and *P. aeruginosa* (74.83%) (Figure 5B). These results indicated that the strain SOIR-1 had the highest and lowest predatory activity against *S. dysenteriae* and *P. aeruginosa*, respectively. 

***Factors involved in the predation by Bdellovibrio bacteriovorus strain SOIR-1***

**Figure 6:**
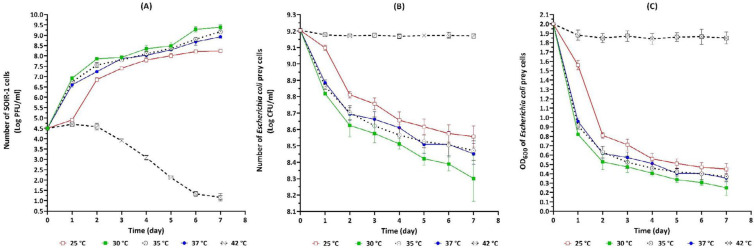
The effect of temperature on the growth and lytic activity of *Bdellovibrio bacteriovorus* strain SOIR-1 indicated by changes in the PFU/ml (A), CFU/ml (B), and OD_600_ (C) parameters in the broth co-cultures (pH 7.4)

The strain SOIR-1 had the predatory activity at 25, 30, 35, and 37 ^°^C. There was little difference in the final number of produced strain SOIR-1 progenies (log PFU/ml) at 30, 35, and 37 ^°^C (9.38±0.14, 9.17±0.08, and 8.92±0.07, respectively) (*P*-value˃0.05). The most intense growth rate of strain SOIR-1 at 30, 35, and 37 ^°^C occurred within one day (*P*-value˃0.05). At 25 ^°^C, however, the strain SOIR-1 required 2 days to reach the most intense growth rate. It means that the growth of strain SOIR-1 at 25 ^°^C was slower than 30, 35, and 37 ^°^C (*P*-value≤0.05). Unlike *E. coli* prey cell, the number of strain SOIR-1 cells gradually decreased at 42 ^°^C (*P*-value≤0.05). In other words, strain SOIR-1 was dying at 42 ^°^C, and no lytic activity was observed at this temperature (Figure 6A). The growth of strain SOIR-1 was directly related to the decrease in the prey number (*P*-value≤0.05). The most intense reduction rate in OD_600_ (and consequently CFU/ml) of prey occurred within one day at 30, 35, and 37 ^°^C (*P*-value˃0.05). It took 2 days at 25 ^°^C to reach the same reduction rate. On the last day of evaluation (day 7), the reduction rates in OD_600_ of prey suspension at 30, 35, and 37 ^°^C were 1.75±0.08, 1.63±0.05, and 1.64±0.06, respectively. No significant decrease in OD_600_ of prey was observed at 42 ^°^C (*P*-value>0.05) due to the death of strain SOIR-1 (Figures 6B-C).

**Figure 7:**
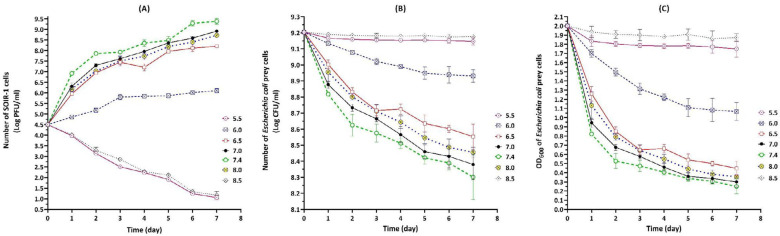
The effect of pH on the growth and lytic activity of *Bdellovibrio bacteriovorus* strain SOIR-1 indicated by changes in PFU/ml (A), CFU/ml (B), and OD_600_ (C) parameters in the broth co-cultures (30 ^°^C).

The predatory activity of strain SOIR-1 was recorded at pH range of 6.0, 6.5, 7.0, 7.4, and 8.0. The strain SOIR-1 gained its highest growth rate at pH 7.4 within one day, while more days were required to reach its maximum growth rate at other pH points. On the last day of evaluation (day 7), the maximum number of produced strain SOIR-1 progenies (log PFU/ml) was 6.1±0.1 (pH 6), 8.2±0.06 (pH 6.5), 8.91±0.06 (pH 7.0), 9.38±0.14 (pH 7.4), and 8.71±0.1 (pH 8.0) (Figure 7A). Compared with other pH points, the most reduction in the CFU/ml and OD_600_ of prey suspension occurred at pH 7.4 (*P*-value≤0.05) (Figures 7B-C). No growth was observed for strain SOIR-1 at pH 5.5 and 8.5. The OD_600_ and CFU/ml of prey suspension were almost unchanged (*P*-value>0.05) at pH 5.5 and 8.5, indicating that the prey cells were able to survive under these conditions. However, the gradual and significant decrease in the number of strain SOIR-1 cells (PFU/ml) showed their sensitivity and death at pH 5.5 and 8.5 (*P*-value≤0.05) (Figures 7A-C). In agreement with the other reports (24), strain SOIR-1 grew between 25-37 ^°^C and pH 6.0-8.0, with the highest predatory activity at 30 ^°^C and pH 7.4.

**Table 1:** The bacterial strains used in this study and their sensitivity to the predation by isolated *Bdellovibrio bacteriovorus* strain SOIR-1

**Species/strains**	**Strain information**	**Source ** ^(a)^	**Lysis ** ^(b)^ **by strain** **SOIR-1**
*Escherichia coli*	Enteropathogenic *E. coli* O111: K58	PTCC 1270	Yes
*Klebsiella pneumoniae*	Capsular serotype 3, isolated from human pneumonia	PTCC 1290 (NCTC 5056)	Yes
*Pseudomonas aeruginosa*	Isolated from wound	PTCC 1310 (CIP A22)	Yes
*Shigella dysenteriae*	Serotype 6	PTCC 1188	Yes
*Salmonella typhi*	-----	PTCC 1609	Yes
*Proteus vulgaris*	-----	PTCC 1079	Yes
*Staphylococcus aureus *subsp.* aureus*	Rosenbach	PTCC 1337 (ATCC 29737)	No
*Staphylococcus saprophyticus *subsp*. saprophyticus*	Type strain, isolated from urine	PTCC 1440 (ATCC 15305) (DSM 20229)	No
*Bacillus subtilis *subsp.* subtilis*	Type strain, Marburg strain	PTCC 1720 (ATCC 6051) (DSM 10)	No
All heat-killed prey inoculated with strain SOIR-1	-----	-----	No
All prey inoculated with 0.22 µm filtered strain SOIR-1	-----	-----	No

^(a)^ PTCC (Persian Type Culture Collection, Tehran, Iran), ATCC (American Type Culture Collection, Manassas, Virginia, USA), NCTC (National Collection of Type Cultures for bacteria, Central Public Laboratory Service, London, UK), CIP (Collection of Institute Pasteur, Paris, France), DSM (Deutsche Sammlung von Mikroorganismen, Braunschweig, Germany)

^(b)^ Indicated by plaque formation through double-layer agar plating technique

**Table 2:** The PCR oligonucleotide primers used in this study for molecular identification of isolated BALO

**Primer name**	**Primer sequence (5ʹ to 3ʹ)**	**Target**	**Specificity**	**Amplicon size (base pair)**
Forward: 63F	CAGGCCTAACACATGCAAGTC	16S rRNA gene	Universal (bacteria)	~1300 (bp)
Reverse: 1378R	CGGTGTGTACAAGGCCCGGGAACG			
				
Forward: Bd529F	GGTAAGACGAGGGATCCT	16S rRNA gene	*Bdellovibrio*-specific	~480 (bp)
Reverse: Bd1007R	TCTTCCAGTACATGTCAAG			
				
Forward: Hit-FW	GACAGATGGGATTACTGTCTTCC	*hit* locus	*Bdellovibrio*-specific	~910 (bp)
Reverse: Hit-RW	GTGTGATGACGACTGTGAACGG			

## Discussion

The most complete data on the isolation of bdellovibrios from Iran were presented in this study through the plaque-based assays (Figure 1B, Supplementary Figure 1), transmission electron microscopy (TEM) (Figure 1C), *Bdellovibrio*-specific PCRs targeting the 16S rRNA gene and *hit* locus (Figure 2), and phylogenetic analysis of 16S rRNA gene (Figure 3). The results indicated that the isolated BALO was a strain of *B. bacteriovorus*, and we designated it as *B. bacteriovorus* strain SOIR-1. We did not examine the predatory mechanism of strain SOIR-1. However, since the strain SOIR-1 had a close phylogenetic relationship with other *B.* *bacteriovorus* strains (Figure 3), we conclude that the strain SOIR-1 would also predate through the periplasmic invasion approach. Furthermore, the genus* Bdellovibrio *comprises two identified species; *B. exovorus *and* B. bacteriovorus*. *B. exovorus* preys on* Caulobacter crescentus* through the epibiotic mechanism of predation, without any periplasmic growth phase (35). The strain SOIR-1 had no close phylogenetic relationship with strains of *B. exovorus *(Figure 3).

The isolation of new *B. bacteriovorus* strain from the rhizosphere soil sample in Iran agrees with the other reports that bdellovibrios are present in diverse terrestrial and aquatic habitats (10). The population levels of *B. bacteriovorus* follow the Lotka-Volterra prey-predator oscillation, where a reversible plastic phenotypic resistance in the prey cells balances the *B. bacteriovorus* expansions. Therefore, although bdellovibrios are present in almost all environmental ecosystems and constitute about 80% of the soil BALOs community, they rarely form numerically dominant populations (32, 36, 37). It has been suggested that BALOs have an ecological balancer role in biological ecosystems (8, 12). Recently, bdellovibrios have been considered as living antibiotics or a source of new antimicrobial agents (11, 17, 38). However, their successful isolation from environmental samples is difficult due to the dependence of the isolation procedures on the appropriate prey and the physicochemical conditions.

Plaque-based assays (Figure 1B, Supplementary Figure 1), as well as monitoring of lysis parameters (OD_600_, CFU/ml, and PFU/ml) in the broth co-cultures (Figure 4, Supplementary Figure 2), were used for evaluation of the bacteriolytic activity of strain SOIR-1. Strain SOIR-1 showed different killing rates toward various prey (Figure 5). This behavior of bdellovibrios is called preferential predation, which reflects their predation efficiency. Preferential predation by bdellovibrios has also been documented before. A study showed that the predation by* B. bacteriovorus *109J is not random, but it infects and kills different prey in a preferential manner. They suggested that predation efficiency is a function of attachment efficiency (39). Li *et al*. stated that BALOs have a prey preference toward prey that were used for their isolation (40). However, this hypothesis was ruled out in this study since we used *E. coli* as prey for isolating the strain SOIR-1, but its highest predation efficiency was toward *S. dysenteriae *(Figure 5). Although the wide prey range toward Gram-negative bacteria is a common feature of *B. bacteriovorus* strains, it has been shown that even closely related strains of *B. bacteriovorus *have different predation efficiencies against the same prey strain, and closely related strains of the same species can be differentially preyed upon by the same *B. bacteriovorus *strain (16, 41, 42). These observations indicate that the interaction between bdellovibrios and prey is not just an ordinary random collision event, and some complex and unknown mechanisms are likely involved in the attachment of bdellovibrios to their preys.

The potential specific receptors on the prey cell surface and their accessibility are likely involved in the attachment and preferential predation by bdellovibrios. Although these receptors are not yet fully identified, there is evidence that they are presumably located in the core portion of lipopolysaccharide (LPS) within the prey cell wall (43). The wide prey range of *B. bacteriovorus *strains can be due to two hypothetical factors; firstly, bdellovibrios can recognize diverse receptors on the surface of various prey cells; these receptors may have different binding affinities. Secondly, bdellovibrios recognize a common motif on the surface of different prey cells as the receptor which is essential for the viability of prey cells (21, 38). The latter hypothesis expresses a kind of survival strategy, i.e., it is not rational for bdellovibrios to use just a simple receptor which can easily lead to prey cell resistance by mutations, unless it is essential for the viability of prey cells. In terms of receptor accessibility, it has been reported that S-layers protect prey cells from predation by bdellovibrios. S-layers may block the access of bdellovibrios to the potential receptors located in the prey cell wall. However, bacterial capsules are not protective barriers against bdellovibrios predation (44, 45). In this study, strain SOIR-1 was also able to lyse *K. pneumoniae*, a bacterium with a typical capsule.

The broad bacteriolytic spectrum of *B. bacteriovorus *strain SOIR-1 reflects its high potential for controlling pathogenic *Enterobacteriaceae* as a living antibiotic (Figure 5). Furthermore, bdellovibrios may act as probiotics to stabilize intestinal microflora because they have recently been isolated from the guts of mammals (15, 32). The predatory ability of strain SOIR-1 at 25-37 ^°^C (Figure 6) and pH 6.0–8.0 (Figure 7) reflects its proper potential for environmental purposes such as *in situ* biocontrol of zoonotic or phytopathogenic Gram-negative bacteria. The lytic activity at 37 ^°^C, as well as in a wide range of pH, are two important advantages in the potential clinical use of strain SOIR-1 as a living antibiotic.

*B. bacteriovorus* was found in the gut of healthy individuals, and this is the most reliable evidence supporting its safety and putative probiotic role in humans (32). There are also no reported incidents for the invasion of mammalian cells by bdellovibrios (17, 20, 46). Furthermore, bdellovibrios have a unique lipid A structure in their LPS, which is much less immunogenic than other bacterial LPS and exhibits lower binding affinity to the LPS receptors presented in the surface of human cells (47). No deleterious side effects have been identified following topical application, ingestion, and injection of *B. bacteriovorus* into vertebrates (11, 48-50). These findings are promising signs which support the hypothesis that predatory bacteria, especially bdellovibrios, might be part of future therapeutic strategies as probiotic, biocontrol agents, or living antimicrobials, especially in the age of growing antibiotics resistance (7, 17, 27, 28, 32, 51, 52).

The term “amphibiotic” represents the dual probiotic and antibiotic nature of bdellovibrios. (11). However, there are still some challenges regarding the use of bdellovibrios as amphibiotic that need to be further addressed. Firstly, predation by bdellovibrios does not completely eradicate the prey population, i.e., there are still some prey cells that have not been invaded. Such transient resistance results from the dynamic parameters of prey and predator and is due to a plastic phenotype rather than a permanently genetically encoded one. So, the resistance to the bdellovibrios is lost quickly (36). This means that bdellovibrios presumably employ a prey’s cellular component in their predation process which is essential for the prey’s viability, and the prey will always be prone to predation. Furthermore, the remaining un-predated cells might be controllable by other complementary management strategies as adjuvants such as antibiotics, enzymes, and bacteriophages (53, 54). Moreover, the quorum-sensing networks between pathogenic bacterial cells may play an important role in their pathogenesis. We have this hypothesis that bdellovibrios can also indirectly suppress the pathogenesis of target bacteria through disrupting quorum sensing networks by killing at least a significant part of the pathogenic population cells. Secondly, compared with bacteriophages, bdellovibrios show broad and non-specific prey range and can attack Gram-negative bacteria from different genera. At first glance, this can be a useful feature when mixed bacterial species cause the infections. However, their effects on the beneficial microbiota should be taken into more consideration (11).

## Conclusion

To the best of our knowledge, this is the first report on the isolation of one *Bdellovibrio* strain with broad and high bacteriolytic activity from Iran. The biological characterization of isolated native strain (SOIR-1) was performed using electron microscopy, specific PCR-based assays, 16S rRNA gene sequence analysis, and bacteriolytic activity. The isolated strain SOIR-1 had the most predatory activity at 30 ^°^C and pH 7.4. The successful lysis of pathogenic *Enterobacteriaceae* nominates strain SOIR-1 as a promising bio-agent for the control and treatment of infections caused by these pathogens. Differential predation by bdellovibrios is a phenomenon that was also shown in this study and indicates that the interactions between bdellovibrios and prey are not just an ordinary random collision or completely receptor-based, but can involve very complicated procedures. The results of this research reject the previous findings claiming that bdellovibrios have the highest predation efficiency toward prey used for their isolation. Due to the heterogeneity of bdellovibrios, further isolation and characterization are necessary to expand the BALOs database. Given the lack of complete and sufficient information on the isolation of bdellovibrios from Iran, this study is a pioneer in itself and pursues the ultimate goal of using them for therapeutic purposes.
